# HA: An Influential Node Identification Algorithm Based on Hub-Triggered Neighborhood Decomposition and Asymmetric Order-by-Order Recurrence Model

**DOI:** 10.3390/e27030298

**Published:** 2025-03-13

**Authors:** Min Zhao, Junhan Ye, Jiayun Li, Yuzhuo Dai, Tianze Zhao, Gengchen Zhang

**Affiliations:** Beijing Key Laboratory of Network System Architecture and Convergence, Beijing University of Posts and Telecommunications, Beijing 100876, China; zhaomin@bupt.edu.cn (M.Z.); li_jiayun@bupt.edu.cn (J.L.); dyz@bupt.edu.cn (Y.D.); zhaotz@bupt.edu.cn (T.Z.); buptzgc@bupt.edu.cn (G.Z.)

**Keywords:** power network, influential node, neighborhood decomposition, order-by-order recurrence model

## Abstract

In recent years, the rise of power network security incidents caused by malicious attacks has drawn considerable attention to identifying influential nodes in power networks. Power networks are a special class of complex networks characterized by a high relative clustering coefficient, which reflects a more intricate connection between nodes. This paper proposes a novel node influence evaluation algorithm based on hub-triggered neighborhood decomposition and asymmetric order-by-order recurrence model. First, the concepts of network directionalization strategy and hub-triggered neighborhood decomposition are introduced to distinguish the functional differences among nodes in the virus-spreading process. Second, this paper proposes the concepts of infected and infecting potential, then constructs a calculation model with asymmetric characteristics based on the order-by-order recurrence method to fully use the information in the connection structure of the adjacent neighborhood. Finally, the influence of the hub node is evaluated by integrating the infected potential and infecting potential of neighbors of multiple orders. We compare our method with the traditional and state-of-the-art algorithms on six power networks regarding Susceptible–Infected–Recovered (SIR) correlation coefficients, imprecision functions, and algorithmic resolution. The experimental results show that the algorithm proposed in this paper is superior in the above aspects.

## 1. Introduction

As power systems continue to evolve, power network informatization levels have increased significantly. This evolution enables the power network to achieve flexible scheduling and intelligent decision-making capabilities but also introduces potential information security risks. Information systems can be exploited by attackers to disrupt power networks through malicious cyber-attacks. In the famous “Venezuela blackout” incident [1], the Venezuelan government claimed that its hydroelectric power plant’s computer system center was under cyber-attack, resulting in a major blackout in 18 states, including the capital of Venezuela. Other incidents, such as the “Ukrainian power network attack” [2] and the “malware infection of the intranet of an Indian nuclear power plant” [3], involved cyber-viruses that attacked critical nodes of the power network, leading to severe social repercussions. The influential node is a class of special nodes in malicious cyber-attacks. When these nodes become infected by a computer virus, they can quickly disseminate the virus throughout the entire network, making them prime targets for attackers. Therefore, identifying the influential nodes in information diffusion has emerged as a significant focus in power network security.

Complex network theory has proven to be a useful tool for studying influential nodes. It employs network characteristics to assess node influence. The network characteristics utilized by algorithms vary depending on the perspectives of researchers. The researches on network topology are mainly divided into three categories, the global-information-based algorithms, the local-information-based algorithms, and the hybrid-information-based algorithms. Global-information-based algorithms utilize the entire network structure, such as the shortest distance between any two nodes, to evaluate the influence of the nodes. Traditional algorithms include the betweenness centrality algorithm [4], the closeness centrality algorithm [5], and the K-shell algorithm [6]. Algorithms developed in recent years include the Gravity Model (GM) algorithm [7,8] and its improved versions such as the Degree K-shell Gravity Model (DKGM) algorithm [9] and Multi-Characteristics Gravity Model (MCGM) algorithm [10]. The quasi-Laplacian algorithm [11] argues that the spectral changes in graphs can be utilized to identify the influential nodes and the importance of nodes is evaluated by the change in quasi-Laplacian energy before and after node removal. Super-Laplacian algorithm [12] extends the algorithm based on Laplacian energy to interdependent networks. Local-information-based algorithms focus on the neighborhood attributes of nodes. Traditional algorithms include degree centrality algorithm [13], clustering coefficient algorithm [14], and eigenvector centrality algorithm [15]. Algorithms developed in recent years include the Improved Information Entropy (IIE) algorithm [16], which jointly considers the propagation rate and information entropy; the H-Index Centrality (HIC) algorithm [17], which considers potential edge weights and thus evaluates the importance of node-connected edges; and the nearest Neighborhood Trust PageRank (NTPR) algorithm [18] based on the structural attributes of the neighbors and the nearest neighbors of the nodes. The Gravity Model and relative Path Combined (GPC) algorithm [19], which takes into account the specificity of paths and nonshortest paths beyond the shortest path, it can be regarded as a node identification algorithm that combines structural and communicative properties. Hybrid information-based algorithms synergize the local and global metrics, commonly found in TOPSIS algorithms that deal with multi-attribute decision-making, such as the Communication Probability and Relative entropy TOPSIS (CPR-TOPSIS) algorithm [20], which identifies influential nodes in complex networks from the view of global, local and location information dimensions, and the TOPSIS-PageRank algorithms [21], which use the TOPSIS algorithm to model the characteristics of the node itself and the PageRank algorithm to measure the interdependence of all nodes to calculate the information entropy of the nodes to evaluate the node importance.

The algorithms utilizing global information, such as the betweenness centrality algorithm and the closeness centrality algorithm, usually have very high time complexity. This time-consuming nature renders them unsuitable for large-scale power networks. The algorithms based on local information usually simplify the characteristics of the nodes to their static parameters and simplify the complex path from the neighbors to the hub node to their distance. This simplification has several problems when identifying the influential nodes: (1) In the virus-spreading process, the same node may demonstrate varying characteristics depending on which node is designated as the initial infected node. However, the static parameters of a node are determined by the network topology. So, the variance in the characteristics of a node in different virus-spreading processes cannot be flexibly captured by static parameters. (2) The spread of the virus from the initial infected node to its higher-order neighborhood is a complex order-by-order process. The infection status of the lower-order neighborhood affects the infection status of the higher-order neighbor. Therefore, the virus-spreading process is closely related to the connection structure between adjacent order neighborhoods. However, the simplification isolates the influence of neighbors of each order on the hub node, and the connection relationships between neighbors of adjacent order are overlooked. The existing local algorithms’ shortcomings limit their effectiveness in finding influential nodes in power networks. Therefore, it is necessary to study the influential nodes in the power network from a new perspective of the effect of virus-spreading trend and adjacent order neighborhoods connection.

To overcome the shortcomings of the existing algorithms, this paper proposes a novel node influence evaluation algorithm based on Hub-triggered neighborhood decomposition and Asymmetric order-by-order recurrence model for power network (HA). (1) We model the power network as a directed network based on the trend of virus spreading. On this basis, we propose the concept of the hub-triggered neighborhood decomposition to distinguish the asymmetric role of different types of neighbors of the initial infected node in virus spreading. (2) We propose a nonlinear computing model of infected potential based on an order-by-order recurrence method and a linear computing model of infecting potential. We integrate the infected potential and infecting potential of each order of neighbors to obtain the evaluation of the influence of the initial infected node. We compare the algorithm proposed in this paper with six classical and state-of-the-art algorithms on six real power networks. The simulation results show that the algorithm proposed in this paper has significant advantages in accurately evaluating the influence of nodes.

The rest of the paper is organized as follows. Section 2 introduces some related research on existing influential node-identifying algorithms. Section 3 introduces the concept of asymmetric decomposition of neighborhood, infected potential, and infecting potential and proposes a novel node influence evaluation algorithm. Section 4 gives the results of the proposed algorithm and analyzes them in comparison with other benchmark algorithms, and the experimental results are analyzed. Section 5 concludes our study.

## 2. Background

In this section, we introduce some representative influential node identification algorithms based on complex network theory, including three traditional algorithms and three state-of-the-art algorithms. A phenomenon worth our attention is the significant difference in the influence scores of the same node in a network given by different algorithms. That is because different algorithms think from different perspectives about the factors that affect the influence of nodes. Combined with our experimental results in Section 4, we believe that the existing algorithms have limitations in observing the factors affecting the influence of nodes and thus cannot achieve a good influence node identification effect. Therefore, the focus of this paper is to design an influential node identification method suitable for power networks based on correctly mining and utilizing the factors influencing the influence of nodes.

### 2.1. Benchmark Algorithms

In the general influential node identification algorithm, the network is regarded as an undirected unweighted graph $G=\left\{V,E\right\}$, where $V=\left\{v_{1},v_{2},\ldots ,v_{N}\right\}$ is the set of nodes in the network. $E=\left\{e_{1},e_{2},\ldots ,e_{M}\right\}$ is the set of edges in the network. The adjacency matrix of the network is $A={\left[a_{ij}\right]}_{N\times N}$.

(1) $$a_{ij}=\left\{\begin{array}{cc} 1, & v_{i}\;\mathrm{is}\;\mathrm{connected}\;\mathrm{to}\;v_{j}\\ 0, & v_{i}\;\mathrm{is}\;\mathrm{not}\;\mathrm{connected}\;\mathrm{to}\;v_{j}\;,\;\mathrm{or}\;i=j \end{array}\right.$$

#### 2.1.1. Degree Centrality Algorithm

The degree centrality [13] measures the influence of a node by the number of its direct neighbors. The degree of node $v_{i}$ is defined as $d_{i}=\sum_{j=1}^{N}a_{ij}$, where *N* is the total number of nodes in the network. The degree centrality of node $v_{i}$ is defined as

(2) $$DC\left(i\right)=\frac{d_{i}}{N-1}$$

The network average degree is the sum of the degree values of all nodes divided by the total number of nodes *N*, which is defined as

(3) $$\langle d\rangle =\frac{\sum_{i=1}^{N}d_{i}}{N}$$

#### 2.1.2. Betweenness Centrality Algorithm

The betweenness centrality [4] is based on the shortest paths. The betweenness centrality of a node $v_{i}$ is positively correlated with the number of shortest paths through $v_{i}$. Thus, the betweenness centrality of node $v_{i}$ is defined as

(4) $$BC\left(i\right)=\sum_{p\neq i\neq q}\frac{\sigma_{pq}^{i}}{\sigma_{pq}}$$

where $\sigma_{pq}^{i}$ is the number of the connection paths between node $v_{p}$ and $v_{q}$, which pass node $v_{i}$. $\sigma_{pq}$ is the number of all connection paths between node $v_{p}$ and $v_{q}$.

#### 2.1.3. Clustering Coefficient Algorithm

The clustering coefficient [14] describes the degree of aggregation between nodes. The clustering coefficient of node $v_{i}$ is defined as

(5) $$C\left(i\right)=\frac{2e_{i}}{d_{i}\left(d_{i}-1\right)}$$

where $d_{i}$ is the degree of node $v_{i}$, and $e_{i}$ is the number of connected edges between the direct neighbors of node $v_{i}$.

#### 2.1.4. K-Shell Algorithm

The K-shell algorithm [6] decomposes the network by gradually removing nodes from the network and dividing the nodes into different levels. The decomposition rules are as follows. Remove all nodes with the degree of 1 and the edges connected to them. If there are still nodes with the degree of 1 in the network after the removal operation, continue to remove nodes with the degree of 1 and repeat this process until the degree of all nodes in the network is at least 2. The K-shell value of all nodes removed so far is recorded as 1. Then, remove nodes with a degree no greater than 2 in the remaining network until the degree of all nodes in the network is at least 3, and the K-shell value of all nodes removed in this process is recorded as 2. Repeat this process until the entire network is decomposed. The higher the K-shell value, the higher the level of the node.

#### 2.1.5. Improved Information Entropy Algorithm

The Improved Information Entropy [16] adjusts the initial information entropy with the infection rate and the number of neighbors to evaluate the influence of a node, which is defined as

(6) $$IIE\left(i\right)=\sum_{j\in \Gamma_{1}\left(i\right)}\left[1-{\left(1-\beta \right)}^{d_{j}}\right]\left(-p_{ji}\log p_{ji}\right)$$

where β is the infectious rate, $p_{ji}=d_{j}/\sum_{v_{m}\in \Gamma_{L}\left(i\right)}$, *L* is the highest order the algorithm considers. The experimental results show that the algorithm achieves its best performance when L=2, so we set L=2 in this paper when conducting the experiments.

#### 2.1.6. Multi-Characteristics Gravity Model Algorithm

The multi-characteristics Gravity Model [10] is based on the model of the gravity formula. The sum of the degree, the K-shell value, and the eigenvector centrality is regarded as the node’s weight. The shortest path length between two nodes is regarded as the distance between two nodes. The influence of a node is defined as

(7) $$MCGM\left(i\right)=\sum_{l_{ij}\leq R,j\neq i}\frac{\left(d_{i}+k_{s}\left(i\right)+x\left(i\right)\right)\left(d_{j}+k_{s}\left(j\right)+x\left(j\right)\right)}{l_{ij}^{2}}$$

where $d_{i}$ is the degree of node $v_{i}$, $k_{s}\left(i\right)$ is the K-shell value of node $v_{i}$, $x\left(i\right)$ is the eigenvector centrality of node $v_{i}$, $l_{ij}$ is the shortest path length between node $v_{i}$ and node $v_{i}$, and is the truncation radius. The best *R* is considered as $0.5\langle d\rangle$, where $\langle d\rangle$ is the average degree of the network.

#### 2.1.7. HIC Centrality Algorithm

The HIC centrality [17] considers that edge weights should be measured by neighborhood information, location information, and topological structure information. The HIC centrality is defined as

(8) $$W_{ij}=\frac{H\left(i\right)+Iter\left(i\right)}{1+c_{i}}+\frac{H\left(j\right)+Iter\left(j\right)}{1+c_{j}}$$

where *H* is the H-index of nodes in the network, $Iter\left(i\right)$ is the K-shell iteration factor of nodes $v_{i}$, $c_{i}$ is the clustering coefficient of node $v_{i}$. The influence of nodes is obtained by adding the corresponding edge weights of nodes, which is defined as

(9) $$HIC\left(i\right)=\sum_{j\in \Gamma_{1}\left(i\right)}W_{ij}$$

where $\Gamma_{1}\left(i\right)$ is the set of direct neighbors of node $v_{i}$.

Table 1 shows the abbreviation, attributes and type of each algorithm.

## 3. Materials and Methods

To solve the problems of neighborhood parameter simplification and isolated analysis of each order in existing algorithms, this paper proposes a novel node influence evaluation algorithm based on hub-triggered neighborhood decomposition and an asymmetric order-by-order recurrence model.

Before we start this section, we need to clarify the terminology used in this paper to eliminate confusion. Typically, papers that use neighborhood information to identify influential nodes often refer to the node whose influence is evaluated as the “hub node” or “central node”. However, in the context of virus spread, we believe that the term “initial infected node” provides a more intuitive understanding of this concept. Therefore, we use both “hub node” and “initial infected node” interchangeably to describe the node whose influence is evaluated, as both terms convey the same meaning.

### 3.1. Network Directionalization and Hub-Triggered Neighborhood Decomposition

**Figure 1:**
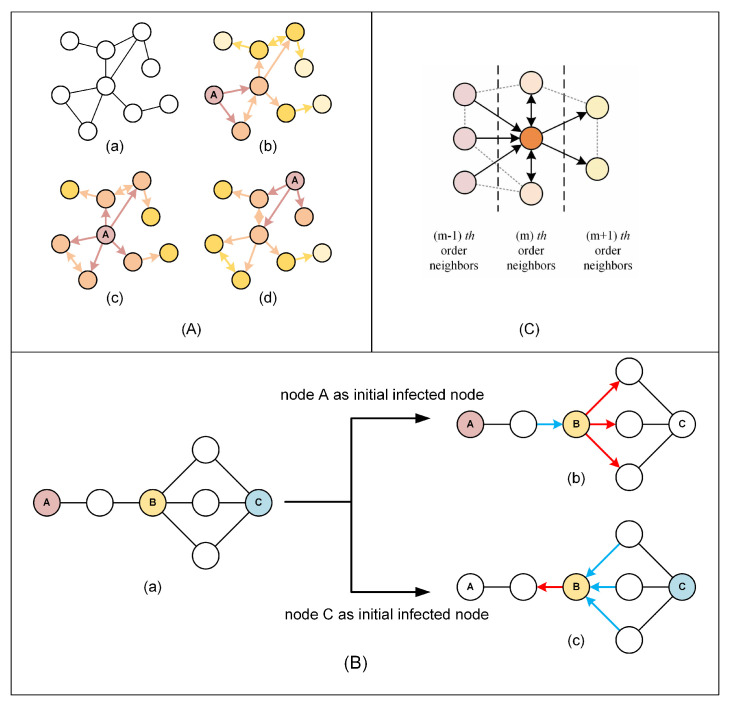
Illustration of how the initial infected node affects the characteristic of nodes in the virus spreading in the network and a method to characterize it: (**A**) (a) An undirected network. (b–d) Infection trend graph obtained from the undirected network shown in Figure 2a with different nodes labeled A as the initial infected nodes. (**B**) Nodes in the network exhibit different characteristics when choosing different nodes as the initial infected node. (a) An original network topology. (b) Characteristics of node B in the virus-spreading process when node A is the initial infected node. (c) Characteristics of node B in the virus-spreading process when node C is the initial infected node. (**C**) Neighborhood asymmetric decomposition. The dark orange node is *m*-th-order neighbor of the initial infected node whose neighborhood is decomposed.

In the previous studies on influential node identification, the power network was generally modeled as an undirected network, as shown in Figure 1A(a). However, the spread of viruses in the network is directional. Figure 1A(b)–(d) show the virus-spreading trend when different nodes are selected as the initial infected node. What brought to our mind is that the same node in a network may exhibit different characteristics when the network has different infection trends. Figure 1B illustrates how the characteristics of an identical node vary depending on the selection of initial infected nodes. The number of nodes attempting to infect node B and the number of nodes that node B attempts to infect are different when nodes A and C are the initial infected nodes. We will discuss later in the following subsection that the number of nodes attempting to infect a node and the number of nodes a node attempts to infect have asymmetric effects on the node’s role in the virus-spreading process. These features cannot be captured when the network is modeled as an undirected network and uses static parameters of the nodes to characterize a node’s influence. Therefore, this paper proposes the concept of hub-triggered neighborhood decomposition to characterize this difference.

Suppose node *c* is the initial infected node; define the set of nodes whose shortest distance from node *c* is *m* as the set of *m*-th-order neighbors of node *c*, denoted as $\Gamma_{m}\left(c\right)$. In particular, $\Gamma_{0}\left(c\right)=\left\{v_{c}\right\}$. All edges connecting different orders of neighbors point from the low-order neighbors to the high-order neighbors. The remaining edges are treated as bidirectional edges. We refer to the resulting network as the infection trend graph of node *c*.

The edges of any node in the network are divided into three types in the infection trend graph: the edges pointing to the node, the edges pointing out of the node, and the edges pointing both to the node and its neighbors of the same order. We refer to these three types of edges as “in-edge”, “out-edge”, and “parallel-edge”, respectively. The neighbor connected to the node by in-edge is called the “in-neighbor”, and the number of in-neighbors is termed the “in-degree”. When node *i* is the initial infected node, node *j* is the *m*-th-order neighbor of node *i*, denote the in-degree of node *j* as $d_{m}^{in}\left(i,j\right)$. Similarly, we can define $d_{m}^{out}\left(i,j\right)$ and $d_{m}^{para}\left(i,j\right)$. The process of distinguishing different types of edges and neighbors of node is called the neighborhood asymmetric decomposition. Figure 1C shows the result of the neighborhood hub-triggered decomposition of an *m*-th-order neighbor of the initial infected node. The term “hub-triggered” indicates that the neighborhood decomposition is a result of the choosing of the hub or the initial infected node.

### 3.2. The Asymmetric Order-by-Order Recurrence Model

**Figure 2:**
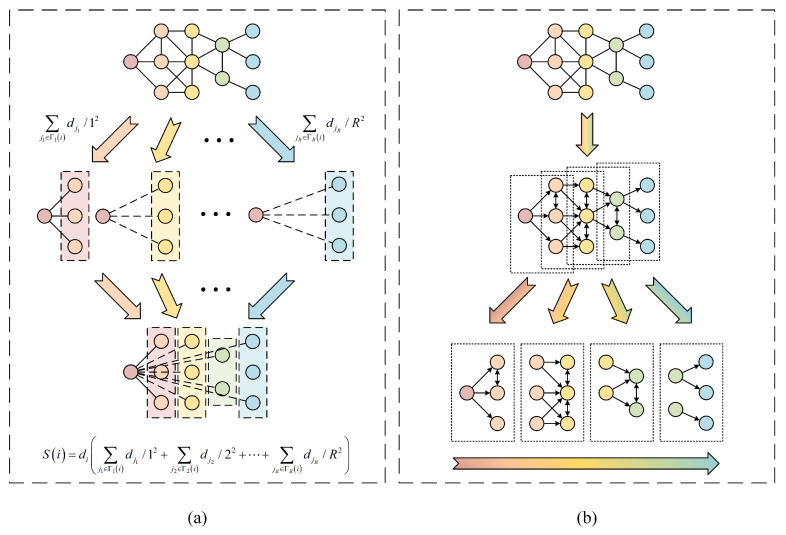
The difference between Gravity Model algorithm and the algorithm proposed in this paper in terms of design ideas: (**a**) The demonstration of the Gravity Model algorithm. (**b**) The order-by-order process of the virus spread.

The existing algorithms tend to consider the effect of each order of neighbors on the central node separately. Take the famous Gravity Model [8], for example, the influence of the hub node is calculated as $S\left(i\right)=\sum_{j\neq i,l_{ij}\leq R}d_{i}d_{j}/{l_{ij}}^{2}$, where $d_{i}$ and $d_{j}$ are respectively the degree of the hub node *i* and its neighbor *j*, $l_{ij}$ is the distance between node *i* and node *j*, *R* is the truncation radius. The characteristics of neighbors are simplified into their degree and distance from the hub node. The neighbors of each order appear to be independent, and the connection between neighbors of adjacent orders is ignored. This utilization of the neighborhood information can be demonstrated in Figure 2a. However, the spread of the virus occurs in an order-by-order manner, as shown in Figure 2a. The infection status of each order’s neighbors is related to the infection status of its lower-order neighbors. Therefore, the influence evaluation model constructed by the existing algorithms misses a large amount of critical information on the connection structure between the adjacent neighborhoods. To address this problem, we propose an order-by-order recurrence model in this paper. We will first introduce the concepts of the “infected potential” and “infecting potential”, followed by a detailed description of how to construct the order-by-order recurrence model.

From the perspective of the infection trend, the in-neighbors and parallel-neighbors of a node are the source of the node’s infection. The larger the in-degree and parallel-degree of a node, the more ways the node can be infected, thus the greater the potential of the node being infected. But before we construct the computational model to characterize a node’s infected potential, let us consider a simple case: a node has *s* infected neighbors, and each neighbor has a probability of χ to infect the node, then the node’s infected probability is $\gamma =\left(1-{\left(1-\chi \right)}^{s}\right)$. It indicates that the infected potential of a node does not increase linearly with its in-degree and parallel-degree. Meanwhile, since the virus transmitting process of a node to each out-neighbor and parallel-neighbor is independent, leading to a linear growth in the infecting potential as the out-degree and parallel-degree increase. The asymmetry of the nonlinear relationship between the in-degree and the infected potential and the linear relationship between the out-degree and the infecting potential further reveal the necessity of the neighborhood hub-triggered decomposition.

On the basis of the analysis above, we now construct the computational model for the node influence. Inspired by $\gamma =\left(1-{\left(1-\chi \right)}^{s}\right)$, we define the infected potential (IDP) of node *i*’s *m*-th-order neighbor node *j* when node *i* is the initial infected node as

(10) $${IDP}^{\left(m\right)}\left(i,j\right)=\left\{\begin{array}{cc} \; & NL\left(1+\frac{1}{2}d_{m}^{para}\left(i,j\right);\chi \right),m=1\\ \; & NL\left(\sum_{a\in \Gamma^{in}\left(j\right)}IDP^{\left(m-1\right)}\left(i,a\right)+\frac{1}{2}\sum_{b\in \Gamma^{para}\left(j\right)}PIP^{\left(m\right)}\left(i,b\right);\chi \right),m>1 \end{array}\right.$$

where $NL\left(s;\chi \right)=\left(1-{\left(1-\chi \right)}^{s}\right)$, $\Gamma^{in}\left(j\right)$, $\Gamma^{para}\left(j\right)$ is the set of in-neighbors and parallel-neighbors of node *j*, respectively. χ is a free parameter. We set χ=0.8 in this paper. $PIP^{\left(m\right)}\left(i,j\right)$ is the “pre-infected potential” of node *j*. Before the node of the same order can infect each other, they must first be infected by their in-neighbors of lower order. We estimate the pre-infected potential of a node using only its in-neighbors. Define the pre-infected-potential of node *j* as

(11) $$PIP^{\left(m\right)}\left(i,j\right)=NL\left(\sum_{a\in \Gamma^{in}\left(j\right)}IDP^{\left(m-1\right)}\left(i,a\right);\chi \right),m>1$$

where $\Gamma^{in}\left(j\right)$ is the set of in-neighbor of node *j*.

Define the infecting potential of node *i*’s *m*-th-order neighbor node *j* as

(12) $$IGP^{\left(m\right)}\left(i,j\right)=d_{m}^{out}\left(i,j\right)+0.5d_{m}^{para}\left(i,j\right)$$

Integrating the infected potential and infecting potential, the spreading potential of node *i*’s *m*-th-order neighbor node *j* is defined as

(13) $$SP^{\left(m\right)}\left(i,j\right)=IDP^{\left(m\right)}\left(i,j\right)\times IGP^{\left(m\right)}\left(i,j\right)$$

Define the sum of the spreading potential of all *m*-th-order neighbors of the initial infected node *i* as the *m*-th-order infection coefficient of node *i*:

(14) $$IC^{\left(m\right)}\left(i\right)=\sum_{j\in \Gamma_{m}\left(i\right)}SP^{\left(m\right)}\left(i,j\right)$$

In particular, since the initial infected node is definitely infected, it can be considered to have an infected potential of 1 and an out-degree equal to its degree. Therefore, we define $IC^{\left(0\right)}\left(i\right)=d_{i}$.

Define the spreading ability of the initial infected node as the sum of the infection coefficients of each order:

(15) $$SA\left(i\right)=\sum_{s=0}^{l}IC^{\left(s\right)}\left(i\right)$$

Generally speaking, considering the third-order neighbors can balance the effectiveness and complexity of the algorithm, so in this paper, we take l=3.

To more fully utilize the information of higher-order neighbors without increasing the complexity as much as possible, the neighborhood spreading ability of nodes is defined as

(16) $$NSA\left(i\right)=\sum_{j\in \Gamma_{1}\left(i\right)}SA\left(j\right)$$

We take the NSA of a node as the influence of the node. Algorithm 1 demonstrates the pseudo-code of the HA algorithm.
**Algorithm 1** HA Algorithm   **Input:** The Adjacency Matrix *A* of the network.   **Output:** The $NSA\left(i\right)$ Value of each node.
1:  **for** each node $v_{i}$ in the network **do**2:        **for** neighborhood order m=1 to 3 **do**3:              **for** each *m*-th order neighbor $v_{j}\in \Gamma_{m}\left(i\right)$ **do**4:                      **if** m=1 **then**5:                            $IDP^{\left(m\right)}\left(i,j\right)=NL\left(1+\frac{1}{2}d_{m}^{para}\left(i,j\right);\chi \right)$6:                      **else**7:                            $PIP^{\left(m\right)}\left(i,j\right)=NL\left(\sum_{a\in \Gamma^{in}\left(j\right)}IDP^{\left(m-1\right)}\left(i,a\right);\chi \right)$8:                            $IDP^{\left(m\right)}\left(i,j\right)=NL\left(\sum_{a\in \Gamma^{in}\left(j;i\right)}IDP^{\left(m-1\right)}\left(i,a\right)+\frac{1}{2}\sum_{b\in \Gamma^{para}\left(j;i\right)}PIP^{\left(m\right)}\left(i,b\right);\chi \right)$9:                      **end if**10:                   $IGP^{\left(m\right)}\left(i,j\right)=d_{m}^{out}\left(i,j\right)+\frac{1}{2}d_{m}^{para}\left(i,j\right)$11:                   $SP^{\left(m\right)}\left(i,j\right)=IDP^{\left(m\right)}\left(i,j\right)\times IGP^{\left(m\right)}\left(i,j\right)$12:           **end for**13:           $IC^{\left(m\right)}\left(i\right)=\sum_{j\in \Gamma_{m}\left(i\right)}SP^{\left(m\right)}\left(i,j\right)$14:     **end for**15:     $SA\left(i\right)=\sum_{m=0}^{3}IC^{\left(m\right)}\left(i\right)$16:     $NSA\left(i\right)=\sum_{j\in \Gamma_{1}\left(i\right)}SA\left(j\right)$17:**end for**


## 4. Results

### 4.1. Data Set and Statistical Characteristics

To verify the effectiveness of the proposed algorithm HA, we conducted experiments on the MATLAB 2024a platform and compared HA’s performance with six algorithms on six power networks. We selected the following networks of different scales as our test cases: Rte 73 [21], IEEE 300 [22], Rte 1951 [23], Goc 2000 [24], Goc 2742 [25], and Power 4941 [26]. Also, we chose the following complex networks to compare with the power network: Karate [27], Dolphins [28], Jazz [29], and Email [30].

Table 2 shows the characteristic parameters of six networks, including the total number of nodes *N*, the total number of edges *M*, the average degree $\langle d\rangle$, the characteristic path length *S*, the average clustering coefficient of the network *C*, the clustering coefficient of a random network $C_{random}$, which has the same number of nodes and edges as the corresponding compared networks and the ratio of *C* to $C_{random}$.

From Table 2, we observe that $C/C_{random}$ is significantly higher in power networks than in most social networks, suggesting that virus spread in power networks is faster, more extensive, and harder to control. Therefore, it is essential to accurately identify the nodes in power networks that play a significant role in virus spread. These nodes require special protection, as they are crucial to the safe and stable operation of power networks.

### 4.2. Simulation Analysis

To validate the performance of HA, we give comparative results of the performance of six well-known algorithms in six real power networks with different topological characteristics. Our main goal is to identify the nodes that significantly impact virus spread.

#### 4.2.1. The Kendall’s Tau Correlation Coefficient with SIR

The SIR model [31] is derived from the infectious disease transmission model, and by finding the total number of infected nodes under different infection probabilities, the transmission ability of nodes in the network is measured. In the SIR model, nodes have three states: susceptible (S), infected (I), and recovered (R). The susceptible node has not yet become infected, but there is a risk of infection. The infected nodes are transformed from susceptible nodes and can infect susceptible nodes. The healing node is transformed from the infected node, which refers to the healed immune individual and will not be infected again. In the initial stage, the selected node becomes the initial infected node, and the infected node infects its neighbors with a certain probability and recovers with a probability *r*. We set r=1 for simplicity [32]. The number of infected and recovered nodes grows until it reaches a steady state. The SIR model simulates the spreading process of the virus in an experimental way, which is closer to the most real virus infectious model, so we believe that the infectious result of the SIR model is the infectious result under actual conditions.

We use the SIR algorithm to simulate the virus-spreading process under a range of infection rates around the epidemic threshold $\beta_{th}=\langle d\rangle /\left({\langle d\rangle }^{2}-\langle d\rangle \right)$ [33,34]. After repeating the SIR algorithm 500 times, we take the average number of nodes recovered as the infectious capacity of nodes. Kendall’s tau correlation coefficient is used to evaluate the consistency between the sorting results of each algorithm and the sorting results of SIR. We compare the node influence ranking given by each algorithm with the ranking of the SIR algorithm to obtain Kendall’s tau correlation coefficient of the two ranking sets. Kendall’s tau correlation coefficient is defined as

(17) $$\tau =\frac{2}{N\left(N-1\right)}\sum_{i<j}sgn\left[\left(x_{a,i}-x_{b,i}\right)\left(x_{a,j}-x_{b,j}\right)\right]$$

where $sgn\left(x\right)$ is the sign function, *N* is the total number of nodes in the network. Giving two sequences A and B, denote $x_{a,i}$ and $x_{a,j}$ as the values of the *i*-th and *j*-th positions of the sequences A, and sequences B likewise. In this paper, the sequences A and B represent the influence score given by the algorithm and the influence score given by the SIR model sorted by node number, respectively. $\left(x_{a,i},x_{b,i}\right)$ is a sequence pair. If $\left(x_{a,i}-x_{b,i}\right)\left(x_{a,j}-x_{b,j}\right)>0$, $\left(x_{a,i},x_{b,i}\right)$ and $\left(x_{a,j},x_{b,j}\right)$ are concordant, if $\left(x_{a,i}-x_{b,i}\right)\left(x_{a,j}-x_{b,j}\right)<0$, $\left(x_{a,i},x_{b,i}\right)$ and $\left(x_{a,j},x_{b,j}\right)$ are discordant. Otherwise, $\left(x_{a,i},x_{b,i}\right)$ and $\left(x_{a,j},x_{b,j}\right)$ are neither concordant nor discordant. The higher Kendall’s tau coefficient, the more the trend of the results of the two algorithms is the same, and the higher the similarity.

**Figure 3:**
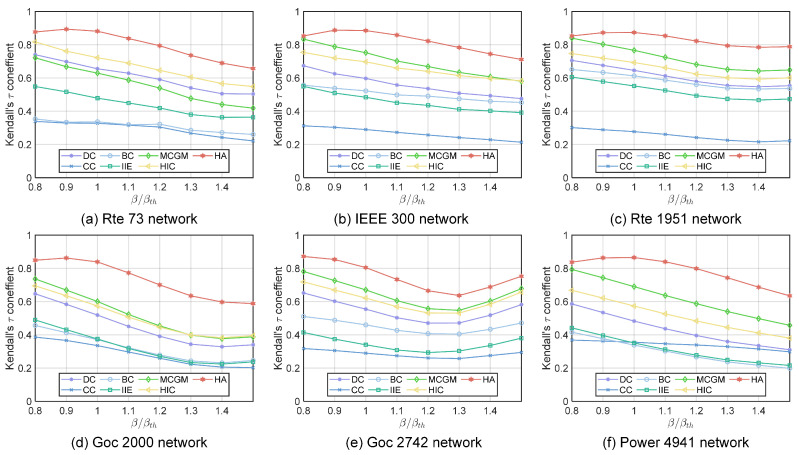
Kendall’s tau coefficient (τ) between the ranking list calculated by six different algorithms and the SIR ranking list with different infection probabilities β. Simulation results of the SIR model are obtained through an average of 500 independent experiments: (**a**) Rte 73 network. (**b**) IEEE 300 network. (**c**) Rte 1951 network. (**d**) Goc 2000 network. (**e**) Goc 2742 network. (**f**) Power 4941 network.

We calculate Kendall’s tau correlation coefficient of the ranking result given by the SIR model under a range of epidemic thresholds and the results given by each algorithm. A higher Kendall’s tau correlation coefficient indicates that the result of the algorithm is closer to the actual virus-spreading process. As shown in Figure 3, the HA algorithm consistently outperforms other algorithms under different infection rates in six power networks. The Kendall tau correlation coefficients of the HA algorithm are greater than 0.8 in all sizes of networks when the infection rate equals the infection threshold. The HA algorithm generally improves Kendall’s tau coefficient by 0.1 to 0.2 compared with the suboptimal algorithm when the infection rate is around or higher than the infection threshold, and it has comparable performance to the suboptimal algorithm when the infection rate is lower than the infection threshold.

#### 4.2.2. The Algorithm Accuracy and Resolution

**Figure 4:**
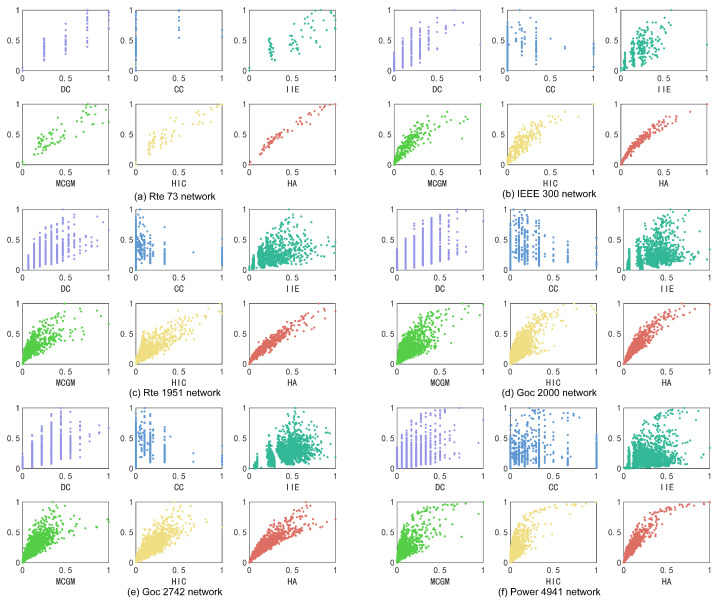
The relevance between the score of the nodes given by algorithms and the infectious capability of the nodes given by SIR experiments. The horizontal axis represents the normalized score of each node. The vertical axis represents the average number of nodes infected by the nodes over 500 SIR experiments normalized: (**a**) Rte 73 network. (**b**) IEEE 300 network. (**c**) Rte 1951 network. (**d**) Goc 2000 network. (**e**) Goc 2742 network. (**f**) Power 4941 network.

To assess the algorithms’ ability to differentiate between various nodes and accurately evaluate their influence, we compare the normalized node influence scores with the infectious capability of the nodes given by the SIR model in the same network, as shown in Figure 4. The vertical axis represents the average number of nodes infected by the nodes over 500 SIR experiments normalized.

The distribution of the scattered points would be synthesized into a straight line or approximately a straight line if the score of the nodes accurately reflects their infectious capabilities. As shown in Figure 4a,c,e, the HA algorithm shows a strong ability to fit a straight line with fewer outlier nodes compared with other algorithms. In Figure 4b,d,f, the HA algorithm can provide a curve approximated to a straight line with better convergence. The scores given by the HA algorithm and the actual infectious ability in all six graphs show a relatively strong linear correlation, proving that the HA algorithm is more effective in expressing the infectious characteristics of the nodes.

In addition, we used the monotonicity [35,36] to quantitively evaluate the rank resolution of the nodes of each algorithm in each network:

(18) $$M\left(R\right)={\left(1-\frac{\sum_{r\in R}n_{r}\left(n_{r}-1\right)}{n\left(n-1\right)}\right)}^{2}$$

where *R* is the ranking vector of nodes, $n_{r}$ is the number of nodes that have the same ranking in the list of *r*, *n* is total number of node ranked. A low level of monotonicity indicates that the algorithm assigns the same score to many nodes and believes they are equally important. However, we must emphasize that the resolution is not directly related to the algorithm’s effectiveness since there are certain nodes in the network that have the same influence. It may not be reasonable to force a distinction between them. Therefore, a good algorithm only needs to guarantee a sufficient resolution. As shown in Table 3, the resolution of the HA algorithm approaches 100%. This proves that the HA algorithm fully utilizes the neighborhood information and can efficiently distinguish nodes based on their influence.

#### 4.2.3. The Top Node Infectious Capability

**Figure 5:**
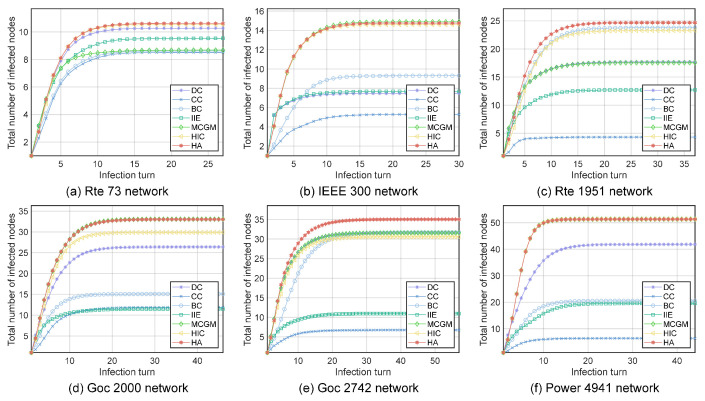
The growth of the infected nodes over time when the top-ranked node obtained by different algorithms is the initial infected node: (**a**) Rte 73 network. (**b**) IEEE 300 network. (**c**) Rte 1951 network. (**d**) Goc 2000 network. (**e**) Goc 2742 network. (**f**) Power 4941 network.

The most influential node exerts the greatest impact on the network once it becomes infected, requiring special concern. We choose the node with the highest scores in each algorithm as the initial infected node and conduct the SIR experiments 500 times. We observe the average number of nodes infected during the virus-spreading process. As shown in Figure 5, the node selected by the HA algorithm outperforms the rest of the algorithms in all six networks, with the largest number of nodes infected with a rapid infection speed. This indicates that the most influential node selected by the HA algorithm is more infectious in the virus-spreading process.

#### 4.2.4. The Imprecision Functions of the Top Nodes

In addition to the most influential nodes, we are also interested in whether the top-ranked set of nodes is correctly identified. We use the imprecision function [6] to evaluate the correlation between the infectious capacity of the top-ranked set of nodes selected by each algorithm and the infectious capacity of the top-ranked set of nodes selected by the SIR model. We compare the imprecision function of the top 2–10% of nodes of each algorithm. We denote the top-scored *x*% node set given by an algorithm as $p_{a}$ and the top-scored *x*% node set given by the SIR model as $p_{s}$, 0<x<100. We denote the number of infected nodes through an SIR experiment when $v_{i}$ is the initial infected node as $\chi_{i}$. The average $\chi_{i}$ of set $p_{s}$ is $\chi \left(p_{s}\right)=\sum_{i\in p_{s}}\chi_{i}/N$. By definition, $\chi \left(p_{s}\right)\geq \chi \left(p_{a}\right)$. The imprecision function is defined as

(19) $$\xi \left(p_{a}\right)=1-\frac{\chi \left(p_{a}\right)}{\chi \left(p_{s}\right)}$$

**Figure 6:**
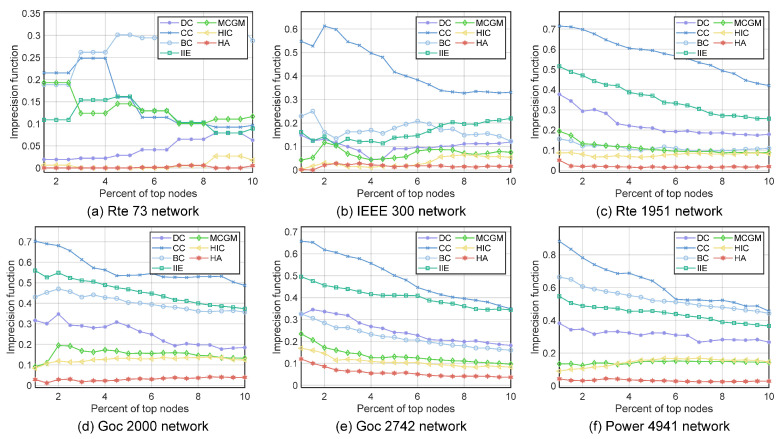
The imprecision function of the top 1% to top 10% top-ranked nodes obtained by different algorithms: (**a**) Rte 73 network. (**b**) IEEE 300 network. (**c**) Rte 1951 network. (**d**) Goc 2000 network. (**e**) Goc 2742 network. (**f**) Power 4941 network.

We perform 500 SIR infection experiments for each node in the $p_{a}$ set as the initial infected node and record the average number of nodes infected by each initial infected node. We start from the $p_{a}$ set, which includes the 2% top-scored nodes, increasing the top-scored nodes included in the $p_{a}$ set by 1% each time, until the $p_{a}$ set includes 10% of the top-scored nodes. As shown in Figure 6, the HA algorithm is able to consistently maintain a very low platform of the imprecision function from the top 1% of nodes to the top 10% of nodes. The imprecision function of the HA algorithm is close to 0 on small to medium-sized networks and below 0.1 in almost every network, while the imprecision function of the suboptimal algorithms goes above 0.1 in a large-scale network. Under the general trend that the imprecision function rises with the increase in the network size, the nonexact function of the HA algorithm in large-scale networks has hardly risen. We argue that the reason why the HA algorithm is able to consistently maintain such a low imprecision function is due to the strong linear correlation between the scores given by the HA algorithm and the actual infectious ability given by the SIR model, as shown in Figure 4. When the score of a node given by an algorithm is a good representation of the node’s influence, the nodes with similar infectious ability will have similar scores. So, even if a slight sorting error happens between the nodes with similar infectious ability, the infectious ability of the two nodes does not have an overly significant difference, since they are comparable in terms of influence. This result indicates that the HA algorithm can reliably find the nodes with the greatest impact on virus spread.

#### 4.2.5. Algorithm Complexity

The time complexity of the algorithm is a crucial metric for the practical use of the power network because the power network sites are often large in number, have a wide range of connections, and keep on expanding. Table 4 lists the time complexity of each algorithm. The average degree $\langle d\rangle$ is usually very small in the power networks, as shown in Table 2; thus, its impact on the complexity is not significant. Table 4 shows that the HA algorithm has a much smaller time complexity compared with the global algorithm BC and has a complexity of the same order of magnitude compared with the local algorithms, which means that the HA algorithm is suitable for large-scale power networks.

## 5. Discussion and Conclusions

A high average clustering coefficient characterizes the topology feature of power networks and makes viruses more likely to spread. To effectively identify the influential node in the power network for further protection, this paper proposes a novel influential node identification method based on hub-triggered neighborhood decomposition and asymmetric order-by-order recurrence model. Through network directionalization and neighborhood decomposition, this paper puts forward a computational order-by-order recurrence model of the infected potential and infecting potential to accurately portray the role of neighbors in virus spreading. In addition, we innovatively use the scatter plots of the influence scores of a node and the number of nodes infected by the initial infected node to evaluate whether the scores given by the algorithms are accurate and reasonable. We conducted experiments on six real power networks and compared them with six benchmark methods in five dimensions to verify the HA algorithm’s validity, reliability, and accuracy. The experiment results show that the HA algorithm is able to consistently outperform the traditional and state-of-the-art algorithms by generally improving Kendall’s tau coefficient by 0.1 to 0.2 compared with the suboptimal algorithm when the infection rates are around or higher than the infection threshold and improving the imprecision by 0.05 to 0.1 compared with the suboptimal algorithm in the large-scale network. We also argue that the strong linear correlation between the scores given by the HA algorithm and the actual infectious ability given by the SIR model contributes a lot to the HA algorithm’s ability to maintain a low imprecision function. The study results have far-reaching and practical guiding significance for power network fault prediction and critical site protection after fault alarms. We look forward to further refining the proposed method in future work and generalizing it to more complex network research areas.

## Figures and Tables

**Table 1:** The the abbreviation, attributes and type of each algorithm. features of different algorithms.

Algorithm	Abbreviation	Attributes	Type
Degree Centrality	DC	Local	Traditional
Betweenness Centrality	BC	Global	Traditional
Clustering Coefficient	CC	Local	Traditional
K-shell	KS	Global	Traditional
Improved Information Entropy	IIE	Local	State of the art
Multi-Characteristics Gravity Model	MCGM	Local	State of the art
HIC Centrality	HIC	Local	State of the art

**Table 2:** Six typical power networks and characteristic parameters.

Network	*N*	*M*	$\langle d\rangle$	*S*	*C*	$C/C_{\mathrm{random}}$
^1^ Rte 73	73	108	2.9589	5.9829	0.0251	0.782
^1^ IEEE 300	300	409	2.7267	9.9353	0.0856	12.2924
^2^ Rte 1951	1951	2373	2.4336	8.9089	0.0409	50.9551
^3^ Goc 2000	2000	2810	2.8100	16.3627	0.0632	57.4545
^2^ Goc 2742	2742	4005	2.9212	15.9794	0.0330	47.1429
^3^ Power 4941	4941	6594	2.6691	18.9891	0.0801	160.2000
Karate	34	78	4.5882	2.4082	0.5706	4.2190
Dolphins	62	159	5.1290	3.3570	0.2590	3.2044
Jazz	198	2742	27.6970	2.2350	0.6175	4.3894
Email	1133	5451	9.6222	3.6060	0.2202	25.9488

^1^ Small or medium-sized power networks with sparser nodes. ^2^ Large power networks with sparser nodes. ^3^ Large power networks with more clustered nodes.

**Table 3:** Monotonicity performance of different algorithms.

Network	DC	CC	BC	IIE	MCGM	HIC	HA
Rte 73	0.524	0.050	0.999	0.920	1.000	0.886	0.999
IEEE 300	0.611	0.171	0.869	0.974	1.000	0.980	0.998
Rte 1951	0.613	0.052	0.722	0.957	0.998	0.947	0.990
Goc 2000	0.634	0.157	0.901	0.970	1.000	0.961	1.000
Goc 2742	0.536	0.077	1.000	0.963	1.000	0.950	1.000
Power 4941	0.593	0.117	0.508	0.965	1.000	0.957	1.000
Mean value	0.585	0.104	0.833	0.958	1.000	0.947	0.998

**Table 4:** Complexity differences between algorithms.

Algorithm Abbreviation	Complexity
DC	$O\left(N\right)$
BC	$O\left(NM+N^{2}\log N\right)$
CC	$O\left(NM\right)$
IIE	$O\left(N\right)$
MCGM	$O\left(N{\langle d\rangle }^{3}\right)$
HIC	$O\left(2N{\langle d\rangle }^{3}+N\langle d\rangle \right)$
HA	$O\left(N{\langle d\rangle }^{4}\right)$

## Data Availability

All relevant data are available at: https://github.com/JohnBurlin/Power-Network-Dataset-HA.git (accessed on 12 September 2023).
